# Traveling waves across scales: Different mechanisms but same canonical computation?

**DOI:** 10.7554/eLife.106753

**Published:** 2025-11-19

**Authors:** Laura Dugué, Frédéric Chavane

**Affiliations:** 1 Université Paris Cité, CNRS, Integrative Neuroscience and Cognition Center Paris France; 2 Institut Universitaire de France (IUF) Paris France; 3 Aix-Marseille Université, CNRS, Institut de Neurosciences de la Timone, UMR7289 Marseille France; Max Planck Institute for Psycholinguistics Netherlands; University of Texas at Austin United States

**Keywords:** traveling waves, cortex, spatial scales, generative mechanisms

## Abstract

Non-stationary dynamical cortical states − neural activity changing on the topology of the cortex across time − and in particular traveling waves, is an emerging topic. In this article, we propose that similar spatio-temporal traveling wave patterns observed across cortical scales are underpinned by generative mechanisms that differ in nature, that we categorize as first- and second-order traveling waves. This original definition provides a unifying framework making testable predictions at both mechanistic and functional levels. While having diverse mechanistic origins, we propose that traveling waves across spatial and temporal scales subserve a canonical computation at the core of a variety of brain functions, thereby ordering neuronal processing to impose a computational syntax.

For nearly a century, fast temporal dynamics of brain signals (sub-second scale) have been investigated, with an emphasis on their stationary properties, such as temporal oscillations with constant frequency over time and constant phase over cortical space. Research has notably focused on their potential link to cognitive functions, including for instance perception, attention, and memory (for review VanRullen, 2016; Kienitz et al., 2022; Buzsáki, 2006). Theories have progressively emerged suggesting that inter-areal brain dynamics can provide a functional framework for information processing. Well-known theories include binding-by-synchrony (Singer and Gray, 1995), communication-through-coherence (Bastos et al., 2015), gating-by-inhibition (Jensen and Mazaheri, 2010), and nested oscillations (Bonnefond et al., 2017). Today, the scientific community has gone one step further and has recognized that these dynamical states can be non-stationary, that is the temporal properties of brain signals can vary over the extent of the brain, creating, for instance, cortical traveling waves when phase varies smoothly over space (Hughes, 1995). Importantly, this spatial non-stationarity can play a role in brain computations (Muller et al., 2018; Alamia and VanRullen, 2023; Ermentrout and Kleinfeld, 2001), and in turn, in cognitive functions. In particular, it was proposed that more of the trial-by-trial variance in behavior could be explained by considering jointly the temporal and the spatial dimensions of brain signals (Fakche and Dugué, 2024). The literature has thus seen a recent surge of, and interest in high-impact publications on traveling waves (Figure 1A). In particular, computational models of traveling waves have the highest publication rate (Figure 1B), suggesting a strong theoretical interest in their existence and computational advantage, to be grounded in solid empirical evidence. A major challenge in the field now is to understand the mechanistic underpinning of traveling waves. As will be demonstrated in the present review article, not all measured traveling waves have the same underlying neural origin, not all can have their dynamics (e.g. speed, direction) modulated by external factors, and worse, not all traveling waves can be unambiguously related to signal propagation across the brain. Without careful consideration of the origin of traveling waves, our ability to efficiently explain and predict their functional role is impaired, and progress is hindered. Our review addresses this gap.

**Figure 1.**
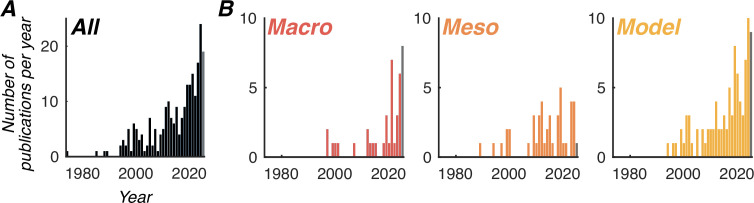
Publication trend on traveling waves (from 1970 to August 2025). (**A**) PubMed search for (travel(l)ing wave(s) OR front propagation OR cortical propagation OR horizontal propagation) AND (cortex) NOT (myosin OR actin). The search was constrained to Title/Abstract. (**B**) Same search constrained to NOT (review). Additional PubMed search terms were added to what we called Macro, Meso, and Model categories (red, dark orange, light orange distributions, respectively). Macro: AND (‘EEG’ OR ‘MEG’ OR ‘electroencephalography’ OR ‘magnetoencephalography’ OR ‘ECoG’ OR ‘electrocorticography’ OR ‘intracranial’). Meso: AND (‘intracellular recordings’ OR ‘single-unit activity’ OR ‘SUA’ OR ‘multi-unit activity’ OR ‘MUA’ OR ‘local-field potential’ OR ‘LFP’ OR ‘two-photon microscopy’ OR ‘multi-electrode array’ OR ‘MEA’ OR ‘voltage-sensitive dye imaging’ OR ‘VSDI’ OR ‘optical imaging’). Model: AND (‘model’ OR ‘computational’). The gray bar in each plot represents the publications in 2025 (from January to August).

Various pitfalls currently slow down the progression of research on the topic. Specifically, one needs to carefully consider that what is called a *traveling wave* may actually originate from very different mechanisms depending on how it was measured and analyzed. Reaching a unifying understanding of traveling waves and their role for brain functions from such a variety of empirical evidence is thus challenging. For instance, when measuring phase propagation using electro- or magneto-encephalography (EEG, MEG), known for their poor spatial resolution, technical and biophysical constraints are impacting potential interpretations of the recorded signal (e.g. source summation, volume conduction, low signal-to-noise ratios Grabot et al., 2025; Petras et al., 2025). Invasive approaches often used to circumvent these issues also have limitations when considering spatio-temporal dynamics. Voltage-sensitive dye imaging (VSDI), for example, is used in non-human primates, carnivores, and rodents (Grinvald and Hildesheim, 2004; Chemla and Chavane, 2010a) to directly measure membrane potential fluctuations at the neuronal population level. Although VSDI has a high spatial and temporal resolution over a large field of view (up to 1–2 cm), the measure is averaging fluctuations across all cellular compartments (neuronal types, glia, neuronal compartments, layers) (Chemla and Chavane, 2010b), thus making it difficult to identify the exact cellular origin of the signal.

Beyond these methodological considerations, the measurement of traveling waves – characterized by an inseparable space-time relation – additionally poses a challenge to current analytical approaches (Muller et al., 2018; Gutzen et al., 2024, Alexander and Dugué, 2024, Alexander et al., 2015). While this is not the focus of the present review, we further discuss this point in the section Evidence for the framework. Here, we argue that non-stationarity in these signals may have different origins, and that they can explain current discrepancies in the literature. We propose an operationalization of traveling waves into first- and second-order, depending on their origin, focusing the current review on sub-second scales (i.e. excluding vasomotion or hemodynamic responses). We then review the evidence in favor of region(s)-dependent integration time constants and conduction delays as explanatory mechanisms underlying both temporal and spatio-temporal neural dynamics reported in the literature and propose a new unifying, across-scale mechanistic principle governing traveling waves. Finally, we review the empirical evidence in favor of this framework and how accounting for task requirements can unify across different empirical findings.

## First- and second-order traveling waves

Researchers can have different views on the exact origin of traveling waves depending on the scale of their observations. While invasive mesoscopic recordings may detect traveling waves (TWs) propagating across the observed network, observations from non-invasive recordings will rather be viewed as emerging from either true long-range axonal propagation or instead from more complex network interactions. Here, we propose an operationalization aimed at clarifying the current state of the literature and reconciling potential inconsistencies, especially regarding the link between certain terminologies and the tools and scales of observations.

*We define a first-order traveling wave as an observable spatio-temporal pattern primarily resulting from the propagation of a transient change of activity between spatially contiguous and interconnected neurons.* The properties of first-order TWs are therefore directly linked to the properties of (1) the corresponding propagation (speed, direction, distance) and (2) the integration time constants of the recipient neurons. Critically, although traveling waves factually exist on all axons of any neuron generating spikes, it is not operationally useful to include in the definition single spikes traveling along a single axon. Instead, we propose that a prerequisite for a traveling wave to be of first order is that the propagating activity arises from a population of neurons producing enough spikes to generate a functionally effective propagating transient of activity. In other words, a first-order traveling wave should be strong enough to significantly modulate information processing by structuring neural population activity in space and in time. This means the TW should induce a change of activity that can be detected. It is important to note that the failure to detect TWs does not necessarily imply their absence altogether. For instance, in the primary visual cortex, the detectability of intra-cortical propagation can depend on the strength of a concomitant feedforward activation, which can be directly manipulated by stimulus contrast (Nauhaus et al., 2009). A first-order TW can also be modulated by ongoing spontaneous activity (Arieli et al., 1996), making it most effectively detected using single-trial detection methods (Alexander et al., 2013; Muller et al., 2014) for both evoked and spontaneous occurrences (Muller et al., 2014; Davis et al., 2020). Furthermore, while TWs are typically identified by an increase in neuronal activity, they can also manifest as net inhibition (Chavane et al., 2000) or as a suppression of activity (Chemla et al., 2019).

The proposed definition of first-order traveling waves mostly constrains their observation to the mesoscopic scale. We categorize here TWs into microscopic (from the neuron up to the column, <200 µm), mesoscopic (from the column up to the extent of a cortical area; up to several cm) and macroscopic TWs (across cortical areas; up to the whole cortex). First-order TWs may potentially exist at the macroscopic scale but are difficult to detect with current recording technologies at that level. Specifically, observing direct feedforward or feedback propagation between a succession of cortical areas is technically challenging because of (1) the high degree of divergence and convergence (Salin et al., 1989) of inter-cortical connectivity, that is neurons contained in a small region send projections to a large region and vice versa, (2) nested feedforward and feedback projections between areas (Wang and Kennedy, 2016), and (3) the importance of recurrent intracortical network in integrating these projections (Douglas and Martin, 2004). Given these considerations, traveling waves observed at larger scales using recording tools such as Local-Field Potential (LFP), EEG, and MEG are effectively more difficult to link to a true propagation of activity along the axons.

*We define a second-order traveling wave as an emergent propagating phenomenon resulting from the intricate interplay of individual propagations within the global network (including feedforward, feedback, and intra-cortical connections*). As a result, a second-order TW does not directly depend on the properties of individual propagations but is an emergent property of their intricate interactions (Budzinski et al., 2023). They manifest as a smooth change in the phase of brain transients (e.g. event-related responses, ERP) or oscillations as a function of their spatial position within the neural network. Specifically, we argue that, because of (1) conduction delays between neuronal populations, within and between brain regions, and (2) integration time constants within each brain region, the same transient activity in one region can generate another delayed transient activity in another region (Abeles, 2012). Consecutive interactions between regions can actually lead to the emergence of multiple cycles of an oscillation (Alamia and VanRullen, 2023). In this case, second-order traveling waves would thus correspond to the gradual change of the phase of these oscillations. Importantly, we will give evidence below that a phase gradient over cortical space is not necessarily driven by the propagation of spikes along long-range axons but can also emerge from, and be controlled by, higher order effects, such as changes in the balance between excitation and inhibition in the network (Alexander et al., 2019, Alexander and Dugué, 2024) or gradients in time constants across cortical regions (Chaudhuri et al., 2015). Note that TW can also be observed in the absence of propagation delays, further supporting this point (Ermentrout et al., 1998; Kang et al., 2023). Critically, the properties of the recorded brain dynamic (e.g. speed) do not necessarily correlate directly with intrinsic neuronal properties (e.g. axonal propagation speed; see some evidence regarding this point in the last section). Similarly, and adding to the complexity of the mechanism, thalamo-cortical propagating loops can affect the phase gradient over the cortical sheet (Alamia and VanRullen, 2023; Alamia and VanRullen, 2019). Second-order traveling waves can thus possibly attain larger scales, from intra- to inter-cortical, and their observation will be constrained by the spatial scale of the observation (from single cell to large population) from various measurement tools (e.g. VSDI, LFP, electro-corticography [ECoG], stereotactic intracranial EEG [sEEG], EEG, MEG). Like *wave groups* in physics, second-order TWs could therefore result from higher-order neuronal interactions at multiple scales for which the underlying mechanisms remain poorly understood.

## A framework for contextualizing potential origins of first- and second-order traveling waves

The term ‘traveling wave’ encompasses a set of processes that can potentially extend beyond the simple propagation of action potentials along an axon. To better apprehend the complexity of a TW, and in particular second-order TW, we propose to use a general framework that demonstrates how only a few parameters can affect the phase of neuronal activity and, as a consequence, the speed of a TW. To illustrate the principle, we describe with a toy model a single transient propagating along the visual system, but the framework is proposed to be generic and can be applied to any interconnected neurons or regions. We show the case of a simple unidirectional propagation within an area (e.g. within V1) and of a feedforward stream (e.g. from area V1 to V2 to V3 to V4) as well as a more realistic recurrent system that includes feedback (e.g. V2 to V1). The aim here is not to be comprehensive but rather to illustrate that the phase of an evoked response does not depend only on the axonal delay from an emitter (e.g. V1) to a recipient structure (e.g. V2). Instead, the response dynamic, and thus the phase, can be strongly influenced by the integrative properties of the recipient structure. The two main steps of integration of action potentials by the recipient structure are first, at the neuronal level, synaptic and dendritic integration, and second, at the micro-circuit level, strong recurrent columnar interactions (Douglas and Martin, 2004). These integrative properties can be globally captured by the so-called *effective time constant* of the recipient structure and the non-linear membrane potential-to-spike transformation. The effective time constant has been introduced in computational neuroscience to account for both the neuronal membrane time constant and the network integrative properties (see Heeger and Zemlianova, 2020, Richardson and Gerstner, 2005; Johannesma, 1968). The latter includes both the dendritic integration time constant and the rapid recurrent interplay between excitatory and inhibitory neurons that will both influence the global neuronal population time constant. The non-linearity of the membrane potential to spike transformation is also crucial, as it will strongly change the shape of the input-output transformation. It is important to note that both the effective time constant and the non-linear transformation are influenced by the neurons’ intrinsic properties (i.e. their biophysics) and the extrinsic network properties (i.e. the anatomy and general state of the local recurrent network).

**Figure 2.**
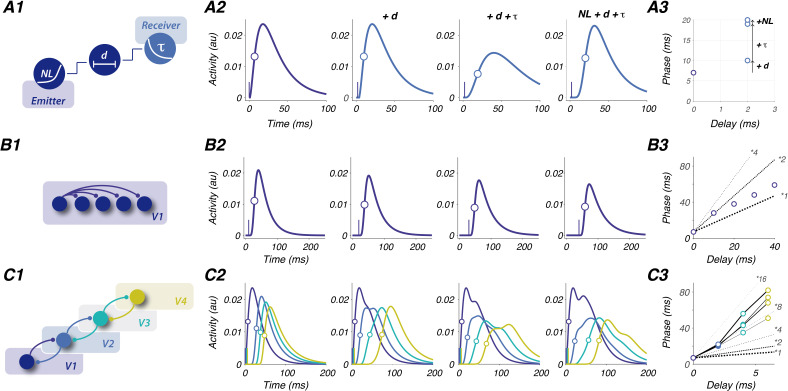
Framework based on region-dependent integration time constants and conduction delays. The visual system is used as a test case. (**A**) General principle. (**A1**) To simulate how a transient activity is transmitted by one emitter and integrated by a downstream recipient structure, we applied 3 transformations: NL, non-linearity due to spike threshold (see Chen et al., 2012; Girard et al., 2001); d, axonal delay (see Girard et al., 2001); and τ, a convolution with a decreasing exponential function of time constant. NL, d, and τ were all estimated from literature (see Table 1). (**A2**) Activity with no transformation (leftmost), and predicted response to the propagation and integration of the V1 transient successively adding up the three parameters (power-law exponent 4 for NL, a delay of 2 ms, and a time constant of 24 ms; inspired from V1-V2 communication). Vertical blue ticks on curves indicate the latency of the V2 transient response. Open circles show half-height time approximating the phase of the transient response. (**A3**) Phases from A2 plotted as a function of delay. (**B**) First-order TW. B1. Intra-area (here V1) propagation along four equally distant positions. (**B2**) V1 response to a transient propagating along the four positions. Vertical blue ticks on curves indicate the latency of the transient response. Open circles show the phase. (**B3**) Phases as a function of delay. (**C**) Second-order TW. (**C1**) Dynamic transformation of a transient along the hierarchical feedforward and feedback interactions between V1-V2-V3-V4 (same color code in B2-B3). (**C2**) Four different steps of transformation of the V1 output transient along V1-V4. From left to right, a single feedforward signal, one feedforward-feedback loop (from V2 to V1, V3 to V2 etc…), two, and three loops. (**C3**) Phases from B2 as a function of delay. Each of the four steps in B2 is connected with lines of increasing widths.

In our toy model, we used the published values of time constants and delays from visual areas V1 to V4 (Table 1) to predict the dynamical transformation of a single transient transferred between areas (Figure 2). Note that the ‘activity’ represented in Figure 2 should be viewed as the sum of synaptic input received by a population of neurons, as seen for instance by VSDI (Chemla et al., 2017) and LFP/EEG (Einevoll et al., 2013). We concentrated on three parameters (Figure 2, A1): (1) the *non-linearity* of the emitting region’s response due to the spike threshold (power law Chen et al., 2012), (2) the *axonal propagation delay* between the two regions (see Table 1), and (3) the *integration time constant* of the recipient structure (Table 1, Figure 2A2). A first simple simulation shows that all three parameters delay the resulting phase of the postsynaptic structure by tens of milliseconds (Figure 2A3). Realistic estimates of these parameters are therefore important for predicting the evolution of phase in various network configurations. Next, we will simulate how the phase of transient stimulation evolves when propagation occurs within intracortical and intercortical networks using region-dependent integration time constants and conduction delays.

**Table 1.** Communication (blue) and integration (green) neuronal space and time constants (within visual cortex). Speeds for communication after V2 were inferred from axonal diameter of connections between V1-V4 (Innocenti et al., 2014; Rockland et al., 1994), V1-MT (Nhan, 2010; Rockland, 1989), V2-MT (Rockland, 1995) and V1-V2 (Rockland and Virga, 1990), using a documented conversion formula (Innocenti et al., 2014). For V4-FEF, the speed was estimated using measured propagation delay and distance reported in corresponding columns. Time constants for V2 were estimated from simulations (Chaudhuri et al., 2015) All other values are extracted from listed references.

	Distance (mm)	Propagation Delay (ms)	Speed (m/s)		Time Constant (ms)	Latency (ms) (Lamme and Roelfsema, 2000) (earliest-mean)
V1-V1	6 (Angelucci et al., 2002)	6–60 (Girard et al., 2001; Muller et al., 2014; Nowak and Bullier, 1997; Reynaud et al., 2012; Grinvald et al., 1994)	0.1–0.5 (Muller et al., 2014; Girard et al., 2001; Nowak and Bullier, 1997; Grinvald et al., 1994; Reynaud et al., 2012)	V1	25–34 (Chemla et al., 2019)	35–72
V1-V2	9.3 Markov et al., 2013)	1 (V1-V2) 1.5 (V2-V1) (Girard et al., 2001)	1–7 (Girard et al., 2001)	V2	34–38 (Chaudhuri et al., 2015)	54–73
V1-V4	14.8 (Markov et al., 2013)	3 (V1-V4) 9 (V4-V1) (van Kerkoerle et al., 2014)	2–6	V4	35–40 (Zeraati et al., 2023)	61–106
V1-MT	12.5 (Markov et al., 2013)	1.2–1.4 (Movshon and Newsome, 1996)	8–11	MT	64–77 (Murray et al., 2014)	39–76
V4-FEF	40 (Markov et al., 2013)	6.5 (Noudoost et al., 2021)	~6	FEF	127–195 (Murray et al., 2014)	43–91

We first tested the model on intra-cortical propagations. Intra-cortical recurrent connections account for about 80% of synapses received by a neuron in cortex (Markov et al., 2011) and play a key role in cortical functions (Douglas and Martin, 2004). We integrated intra-V1 horizontal propagation into the toy model (Figure 2B) taking into account two key properties. First, beyond the short-range scale (i.e. equivalent to a hypercolumn scale Chavane et al., 2011), intracortical connectivity triggers post-synaptic activity that stays subthreshold (no spike emitted Bringuier et al., 1999). Therefore, this propagation can be considered monosynaptic in the sense that the same transmitter broadcasts information to a large intracortical network that is not relayed further. Second, propagation through lateral, unmyelinated intracortical axons is an order of magnitude slower than propagations through intercortical, myelinated axons (Chavane et al., 2011; Bringuier et al., 1999; Girard et al., 2001), thus in the range of integrative time constants. As a result of these properties, our toy model shows that the intracortical, gradual phase change directly reflects axonal propagation speed (Figure 2 B2 and B3). In other words, propagation observed within single brain regions in response to a local neural activation mostly reflects axonal propagation and is thus introduced as *first-order TW*.

We then extended the toy model to inter-cortical propagations, up to V4 (Figure 2 C1). Interestingly, empirical reports (Table 1) show that two of the parameters of interest (delay, time constant) are changing along the hierarchy. First, axonal propagation delays vary depending on the distance between pairs of consecutive regions and direction (V1-to-V2 vs. V2-to-V1). Second, individual regions’ time constants increase along the visual hierarchy, and in general from occipital to frontal regions (Chaudhuri et al., 2015). Importantly, we can also notice that inter-areal delays (<1–10 ms) are smaller than time constants (25–70ms). As a result of these parameter changes, the toy model shows that a single feedforward sweep of information leads to an accumulation of phase delays of several tens of milliseconds along the hierarchy, slower than axonal delays that are <10 ms (Figure 2 C2 left, C3). This observation is congruent with previous literature that reports V1-V2 axonal delays <5 ms (Girard et al., 2001) but differences of visual evoked latencies of about 20 ms (reviewed in Girard et al., 2001; Lamme and Roelfsema, 2000). Together, this highlights that the speed of observed feedforward propagation across several brain regions is only partly influenced by axonal propagation and can be greatly slowed down by the intrinsic properties of both the emitting and the receiving structures.

As highlighted above, axonal delays of inter-cortical propagations are one order of magnitude smaller than integrative time constants. It is thus likely that regions iteratively communicate multiple times within the integrative time frame of a single time constant. The toy model shows that feedback between regions can generate a second delayed transient activity that could change the shape of the original response and sometimes lead to an oscillatory response (Alamia and VanRullen, 2023). Since these oscillatory responses are influenced by the time constants of each region and since time constants increase along the hierarchy, one can predict that their oscillatory frequency decreases along the hierarchy (see next section for corresponding empirical observations; Mahjoory et al., 2020). Depending on the number of feedforward-feedback loops (rows in Figure 2 C2), the shape of the evoked response is also affected, and in particular, the rising slope. This results in the slowing down of the phase of the responses by a factor of up to 16 relative to the speed of axonal propagation. Consequently, time constants, but also inter-areal interactions, are both critical in shaping the evoked responses. We argue that propagation observed between brain areas should not be considered as reflective of axonal propagation, but rather as a gradual phase change, that is a complex emergent property of the network itself, thus called *second-order TW*.

Deliberately oversimplified to illustrate our reasoning, the toy model ignores several key properties of the neural network that will further contribute to modulating the phase latency of evoked transients. Here are a few anatomical examples (specifically regarding the delay parameter) as well as integrative ones (time constant and non-linearity parameters). (1) Although we only considered feedforward-feedback loops between consecutive regions (e.g. V1-V2-V3-V4), connections exist between non-consecutive regions, which further add shorter delays to consider (e.g. V1 projects directly to V3 and V4, V2 to V4, and in turn, V4 projects directly back to V2 and V1, V3 to V1 Van Essen et al., 1992). (2) Inter-areal delays are strongly affected by the high degree of divergence of the feedforward and feedback connections (Salin et al., 1989; Van Essen et al., 1992) of the numerous corticothalamic loops (Cortes et al., 2024; Sherman, 2005; Contreras et al., 1997). (3) Inhibitory mechanisms can shift the balance between excitation and inhibition, which has been shown to strongly affect the network time constants (van Vreeswijk and Sompolinsky, 1996, Murphy and Miller, 2009). (4) The network conductance state, which linearly affects the time constant of the neurons, is itself modulated by several factors (e.g. stimulus-dependent, anesthesia, arousal, task-dependent modulation) (Heeger and Zemlianova, 2020). An important demonstration of this modulation comes from a recent study showing that the attentional state of non-human primates is directly modulating the time constant of V4 neurons (Heeger and Zemlianova, 2020; Zeraati et al., 2023). Finally (5), the spike threshold itself should also be considered as a dynamic parameter that is influenced by the history of the membrane potential (Henze and Buzsáki, 2001; Ulinski et al., 1999). With these limitations in mind, however, our toy model critically illustrates that the phase of the evoked response, and thus the TWs, can be influenced by several anatomical and integrative properties of local to long-range networks.

## Evidence for the framework

Second-order traveling waves’ observation is dependent on multi-scale emergent properties of the network, driven by axonal delays and further modulated by structural and integrative properties at microscopic, mesoscopic, and macroscopic scales. In turn, this means that their properties (i.e. speed, direction) are modulated by higher-order control. Integrated into a more functional framework (note, however, that we do not pretend to make specific claims of the way they are mechanistically generated), we argue that task requirements dictate the time constants and the necessary regions for the task, hence the conduction delays, and thus modulate both dynamical states (temporal frequency) and traveling waves (speed and direction). This statement makes a clear functional prediction: brain functions requiring interactions between regions (to perform the task) are communicating via first- and/or second-order traveling waves.

In this last section, we discuss relevant example results of macroscopic TWs measured in the human brain. It is important to note first, however, an important caveat with the reported TWs using extra-cranial measurement techniques (EEG, MEG). These tools are indeed limited by technical and biophysical constraints, which complicate the localization of the relevant sources of activity (Orczyk and Kajikawa, 2022; van den Broek et al., 1998). Although under tight experimental control, one can recover actual mesoscopic traveling wave activity from extracranial recordings (Petras et al., 2025), several non-wave artifacts can appear in EEG and MEG as TWs, including two spatially discrete dipoles of time-lagged activity (Zhigalov and Jensen, 2023; Orsher et al., 2024) or an artifactual observation of the use of spatial filters (Hillebrand and Barnes, 2011; Lin et al., 2008). Below, the reported estimations of the different TW properties are thus simply assumed to be correct for argument’s sake. Additionally, recent work has shown the importance of considering brain geometry, and in particular structural connectivity, in traveling wave analysis as it appears to affect their direction (Pang et al., 2023; Koller et al., 2024). In conclusion, future research will need to focus on these methodological considerations and will take into account the structure of the considered neural network (see Petras et al., 2025; Alexander and Dugué, 2024, for further discussion).

A first line of evidence in favor of our proposed framework concerns the changes in oscillations’ temporal frequency observed across the different regions of the hierarchy. Critically, as we introduced in the previous section, oscillations are presumably emergent properties of feedforward and feedback interactions between regions. Authors have reported gradients of oscillations’ temporal frequencies across the brain at rest, decreasing from alpha (8–12 Hz) in the occipital regions to theta (4–7 Hz) in more parietal and frontal regions (Mahjoory et al., 2020). This is unlikely due to different local intrinsic rhythms, as alpha and beta (12–30 Hz) oscillations have, for example, been reported for frontal regions (Rosanova et al., 2009; Gaillard et al., 2020). One can instead argue that such gradients reflect properties of functional connectivity between regions (Alamia and VanRullen, 2023), specifically under our framework, with different connection delays and time constants. Interestingly, it was shown that in the presence of such frequency gradients, a wave of activity could be explained by smoothly placed weakly coupled oscillators across the cortical sheet (Ermentrout and Kleinfeld, 2001; Zhang et al., 2018). Additionally, a recent study has shown that attentional demand in a visual search task (as manipulated by target discriminability among the distractors) modulates the frequency of the underlying neural oscillations, that is the stronger the attentional demand, the higher the frequency in the occipital pole going from theta to alpha (Merholz et al., 2022). We argue that the modulation of attention demand by the task constraints can modulate cortical interactions through the network time constants (consistent with previous observation Zeraati et al., 2023) or the non-linearities.

Another line of evidence for the framework concerns the TWs’ properties, and in particular their speed, across the brain hierarchy. A recent review of the studies reporting macroscopic TWs observed in human and non-human primates (Fakche, 2022) showed a large variability and overall range in the observed propagation speeds (although not always calculated with, and potentially not considering, actual geodesic distances). With an average speed of 5.2 m/s, ranging from 0.4 to 20 m/s over cortical distances from 5 to 25 cm, the reported speeds are in the range of the conduction delays along myelinated fibers. However, comparing the results from different studies may be misleading as the TW parameters are directly dependent on their measurements. Interestingly, a study by Shack and collaborators showed in the same study, within the same participants, that the speed of macroscopic alpha TWs closely depended on the task that was performed by the participants (Schack et al., 2003). While the processing of concrete nouns led to the activation of a widespread network and slow propagation speeds, processing abstract nouns was related to a more restricted network with faster alpha TWs. This is in line with our proposal (see Figure 2) and argues in favor of task requirements dictating speed properties of second-order TW. Importantly, and a crucial point of this article, a change of speed of the wave depending on the task constraints necessarily means that a second-order TW is being described, that is emerging from the intricate interplay of individual propagations within the global network (Budzinski et al., 2023). Alternatively, a change of second-order TW’s speed could also be due to the spatial scale of the measurement (for a given temporal frequency, a wave recorded over a large scale will have a higher velocity than a wave recorded over a small scale Alexander et al., 2016). The speed of a first-order TW, however, directly depends on axonal propagation speed and is thus constant. Future investigations should pursue this line of research and systematically test, within individual participants with the same recording method, the link between changes in TW speed at a given temporal frequency, cognitive tasks, and the regions involved.

Interestingly, studies have reported that the propagation speed of mesoscopic and macroscopic oscillatory TWs is positively correlated with stimulus-induced temporal frequency, that is the higher the frequency, the faster the speed (Fakche and Dugué, 2024; Tsoneva et al., 2021). Again, this suggests that such TWs are not of first order, as the speed of axonal propagation is not affected by stimulus conditions. Instead, these observations point to second-order TWs, with complex interactions of first-order TWs emitted at each cycle of the stimulus (Budzinski et al., 2023). The frequency-to-phase relation is not trivial anymore and instead points to a higher-order control of the phase of the underlying oscillation. This is supported by our recent result showing that low-spatial frequencies dominate the cortical phase dynamics as recorded with sEEG in human (Alexander and Dugué, 2024), as well as VSDI results showing that unlike for macroscopic TWs, mesoscopic ones have a constant speed across a wide range of stimulus parameters (Muller et al., 2018; Chemla et al., 2019).

Another interesting TW’s property is its direction, and more generally its shape across the cortical medium. With non-invasive recordings in humans, reported observations concern mainly the antero-posterior propagation axis. In fact, TWs appear to take various shapes including linear, expanding/contracting, and rotating. Such shapes appear to be related to the underlying function. In Denker et al., 2018, the authors show that different mesoscopic phase patterns in (pre-)motor cortices are differentially related to either preparation or execution of movement. Another study similarly found that specific wave shapes correlated with specific states of memory processing (Das et al., 2024). These effects could reflect a local change of first-order TWs to produce functionally relevant second-order TWs. This raises the question of the functional and mechanistic relations between first- and second-order TWs, and thus requires further theoretical and experimental work.

## Conclusion and future directions

In this article, we propose a novel distinction between first- and second-order TW. Compared with first-order TWs, second-order TWs are not under the direct and exclusive control of axonal propagation. Yet, phase gradients are maintained and controlled across cortical space. We argue that first-order TWs are necessary to generate second-order TWs and hypothesize that the phase of the TW is actively controlled to subserve brain functions. Note here that we did not discuss another category of slower TWs (on the order of seconds) reported at the hemodynamic and vasomotion levels (Broggini et al., 2024; Raut et al., 2021; Yousefi and Keilholz, 2021; Gu et al., 2021; Matsui et al., 2016). Further work should focus on whether, and how, these waves can interact with, and further structure, the TWs discussed in the present article.

The novel distinction between first- and second-order traveling waves provides a unifying framework able to make testable predictions at both mechanistic and functional levels. While having diverse neurobiological origins, spatial and temporal brain scales and fields of view, we propose that these different traveling waves constitute a canonical computation that implements a range of brain functions. Specifically, TWs organize neuronal processing in space and time, with local neuronal interactions and between-region interactions sequentially organized and prioritized based on the direction and the speed of TWs. In the visual system, the hierarchical processing of features—such as segments, junctions, shapes, and objects—is not only distributed spatially across the cortex but also temporally sequenced (Lamme and Roelfsema, 2000). This temporal organization ensures that each processing step receives adequate time for accurate feature extraction, which is critical for reliable downstream read-out. Reversing the direction of information flow (e.g. from object to segment) can introduce context dependence (Bar, 2004). Moreover, adjusting the speed or sequence of processing may allow the system to dynamically balance sensory evidence and cognitive priors. The time allocated to each step directly impacts the precision of feature representation, making such adjustments particularly relevant for resolving ambiguous stimuli, like the Kanizsa illusion, for instance. We propose that a canonical function of TWs is to order neuronal processing and impose a computational syntax. While first-order TWs establish this order, second-order TWs possess the unique ability to actively modulate their temporal properties—speed, direction, and shape—thereby enriching computational flexibility. Despite their diverse mechanistic origins, TWs may subserve a canonical computation at the core of numerous brain functions. This proposal should lead to further theoretical progress guiding specific empirical research.

How exactly the intricate interactions between feedforward, intra-cortical, and feedback networks are integrated and shaped by local intrinsic dynamics to produce an observable propagation remains unclear. In the future, we need to systematically characterize the empirical integrative properties of the recorded systems to inform neurobiological models of first- and second-order TWs. Ultimately, we advocate for the field to systematically reevaluate empirical observations in the light of computational theories. For TWs specifically, a systematic report of the TWs’ parameters, for example speed, spatial, and temporal frequencies, will allow building more precise and reliable multi-scale computational models, linking first- and second-order TWs and making testable empirical predictions. We have argued that it is the task, and thus the required cognitive function, which dictates the regions involved in a given process and, in turn, the emergent second-order TWs. To test this hypothesis, one will also investigate the frequency-to-region effect in relation to the type of behavioral task. Together, this would allow disentangling the link between the speed of second-order TWs and the temporal frequency of brain oscillations across different regions. Finally, the idea of a functional segregation between first- and second-order TWs raises more conceptual interrogations. If the propagation of first-order waves traveling over short distances parsimoniously suggests a propagation constrained by the topographic organization of the region, through lateral connections, one could predict that second-order TWs rather propagate across a more complex, functional space. This prediction of our framework requires theoretical and empirical investigations.

## Data Availability

The code to reproduce Figure 2 using the proposed toy-model is available on GitHub, copy archived at Chavane, 2025.
